# Supplementary material for: A microtranslatome coordinately regulates sodium and potassium currents in the human heart
Source: eLife. 2019 Oct 31;8:e52654. doi: 10.7554/eLife.52654 (PMC6867827; doi:10.7554/eLife.52654)
Supplement: Supplementary file 1. — The probes were designed using Stellaris probe Designer software with the following parameters: 18 to 20 nucleotides oligo length, a masking level of 5, a minimum spacing length of 2 nucleotides and a maximum number of probes of 48. Due to the length of the N-terminal specific sequence for hERG1a mRNA, the number of probes used to detect hERG1a is limited to 35. [file elife-52654-supp1.pptx]

## Slide 1
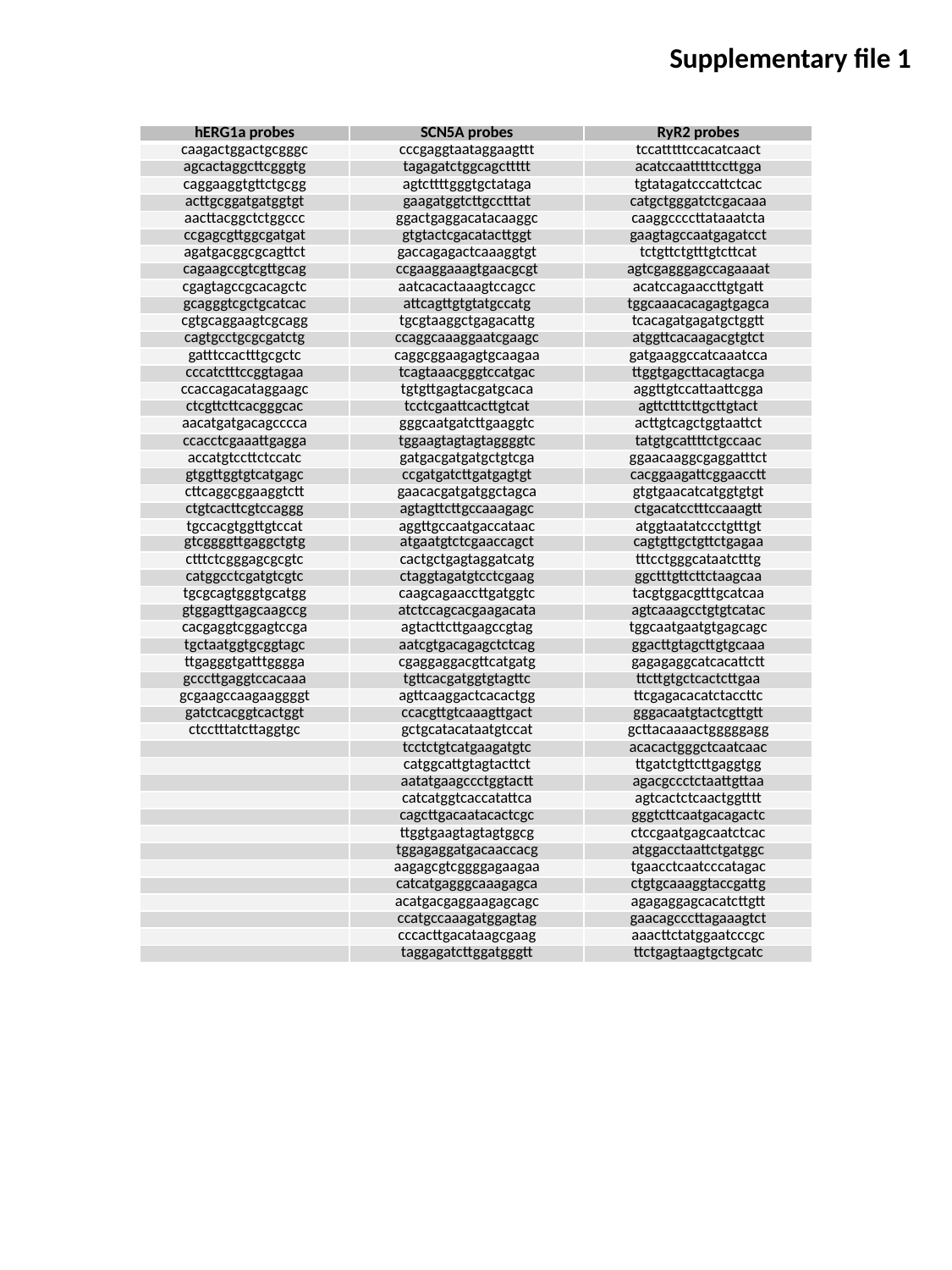

Supplementary file 1
| hERG1a probes | SCN5A probes | RyR2 probes |
| --- | --- | --- |
| caagactggactgcgggc | cccgaggtaataggaagttt | tccatttttccacatcaact |
| agcactaggcttcgggtg | tagagatctggcagcttttt | acatccaatttttccttgga |
| caggaaggtgttctgcgg | agtcttttgggtgctataga | tgtatagatcccattctcac |
| acttgcggatgatggtgt | gaagatggtcttgcctttat | catgctgggatctcgacaaa |
| aacttacggctctggccc | ggactgaggacatacaaggc | caaggccccttataaatcta |
| ccgagcgttggcgatgat | gtgtactcgacatacttggt | gaagtagccaatgagatcct |
| agatgacggcgcagttct | gaccagagactcaaaggtgt | tctgttctgtttgtcttcat |
| cagaagccgtcgttgcag | ccgaaggaaagtgaacgcgt | agtcgagggagccagaaaat |
| cgagtagccgcacagctc | aatcacactaaagtccagcc | acatccagaaccttgtgatt |
| gcagggtcgctgcatcac | attcagttgtgtatgccatg | tggcaaacacagagtgagca |
| cgtgcaggaagtcgcagg | tgcgtaaggctgagacattg | tcacagatgagatgctggtt |
| cagtgcctgcgcgatctg | ccaggcaaaggaatcgaagc | atggttcacaagacgtgtct |
| gatttccactttgcgctc | caggcggaagagtgcaagaa | gatgaaggccatcaaatcca |
| cccatctttccggtagaa | tcagtaaacgggtccatgac | ttggtgagcttacagtacga |
| ccaccagacataggaagc | tgtgttgagtacgatgcaca | aggttgtccattaattcgga |
| ctcgttcttcacgggcac | tcctcgaattcacttgtcat | agttctttcttgcttgtact |
| aacatgatgacagcccca | gggcaatgatcttgaaggtc | acttgtcagctggtaattct |
| ccacctcgaaattgagga | tggaagtagtagtaggggtc | tatgtgcattttctgccaac |
| accatgtccttctccatc | gatgacgatgatgctgtcga | ggaacaaggcgaggatttct |
| gtggttggtgtcatgagc | ccgatgatcttgatgagtgt | cacggaagattcggaacctt |
| cttcaggcggaaggtctt | gaacacgatgatggctagca | gtgtgaacatcatggtgtgt |
| ctgtcacttcgtccaggg | agtagttcttgccaaagagc | ctgacatcctttccaaagtt |
| tgccacgtggttgtccat | aggttgccaatgaccataac | atggtaatatccctgtttgt |
| gtcggggttgaggctgtg | atgaatgtctcgaaccagct | cagtgttgctgttctgagaa |
| ctttctcgggagcgcgtc | cactgctgagtaggatcatg | tttcctgggcataatctttg |
| catggcctcgatgtcgtc | ctaggtagatgtcctcgaag | ggctttgttcttctaagcaa |
| tgcgcagtgggtgcatgg | caagcagaaccttgatggtc | tacgtggacgtttgcatcaa |
| gtggagttgagcaagccg | atctccagcacgaagacata | agtcaaagcctgtgtcatac |
| cacgaggtcggagtccga | agtacttcttgaagccgtag | tggcaatgaatgtgagcagc |
| tgctaatggtgcggtagc | aatcgtgacagagctctcag | ggacttgtagcttgtgcaaa |
| ttgagggtgatttgggga | cgaggaggacgttcatgatg | gagagaggcatcacattctt |
| gcccttgaggtccacaaa | tgttcacgatggtgtagttc | ttcttgtgctcactcttgaa |
| gcgaagccaagaaggggt | agttcaaggactcacactgg | ttcgagacacatctaccttc |
| gatctcacggtcactggt | ccacgttgtcaaagttgact | gggacaatgtactcgttgtt |
| ctcctttatcttaggtgc | gctgcatacataatgtccat | gcttacaaaactgggggagg |
| | tcctctgtcatgaagatgtc | acacactgggctcaatcaac |
| | catggcattgtagtacttct | ttgatctgttcttgaggtgg |
| | aatatgaagccctggtactt | agacgccctctaattgttaa |
| | catcatggtcaccatattca | agtcactctcaactggtttt |
| | cagcttgacaatacactcgc | gggtcttcaatgacagactc |
| | ttggtgaagtagtagtggcg | ctccgaatgagcaatctcac |
| | tggagaggatgacaaccacg | atggacctaattctgatggc |
| | aagagcgtcggggagaagaa | tgaacctcaatcccatagac |
| | catcatgagggcaaagagca | ctgtgcaaaggtaccgattg |
| | acatgacgaggaagagcagc | agagaggagcacatcttgtt |
| | ccatgccaaagatggagtag | gaacagcccttagaaagtct |
| | cccacttgacataagcgaag | aaacttctatggaatcccgc |
| | taggagatcttggatgggtt | ttctgagtaagtgctgcatc |
