# Supplementary material for: A microtranslatome coordinately regulates sodium and potassium currents in the human heart
Source: eLife. 2019 Oct 31;8:e52654. doi: 10.7554/eLife.52654 (PMC6867827; doi:10.7554/eLife.52654)
Supplement: Supplementary file 2. — The linear correlation between the different combination of mRNAs was evaluated using the Pearson correlation coefficient. Because the Pearson coefficient is highly sensitive to outliers and only assess linear correlation, the Spearman’s correlation coefficient was also calculated. Both tests revealed a significant correlation between hERG1a and SCN5A mRNAs and no significant correlation for hERG1a/RyR2, hERG1a/GAPDH and SCN5A/GAPDH pairs. Levels of significance were adjust with a Bonferroni correction taking into account correlation coefficients and either linear correlation or non-linear correlation for Pearson’s and Spearman’s test respectively. [file elife-52654-supp2.pptx]

## Slide 1
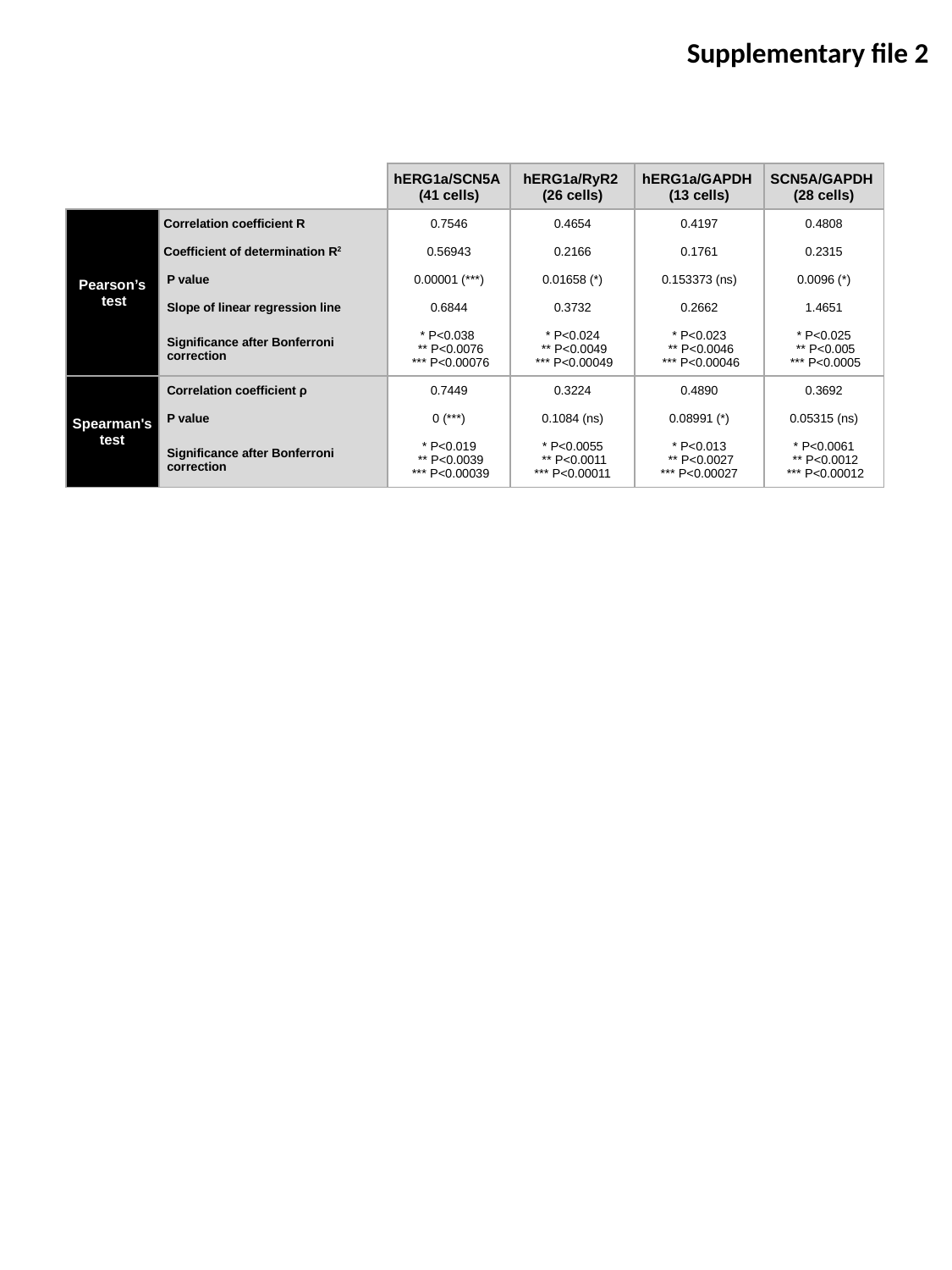

Supplementary file 2
| | | hERG1a/SCN5A (41 cells) | hERG1a/RyR2 (26 cells) | hERG1a/GAPDH (13 cells) | SCN5A/GAPDH (28 cells) |
| --- | --- | --- | --- | --- | --- |
| Pearson’s test | Correlation coefficient R | 0.7546 | 0.4654 | 0.4197 | 0.4808 |
| | Coefficient of determination R2 | 0.56943 | 0.2166 | 0.1761 | 0.2315 |
| | P value | 0.00001 (\*\*\*) | 0.01658 (\*) | 0.153373 (ns) | 0.0096 (\*) |
| | Slope of linear regression line | 0.6844 | 0.3732 | 0.2662 | 1.4651 |
| | Significance after Bonferroni correction | \* P<0.038 \*\* P<0.0076 \*\*\* P<0.00076 | \* P<0.024 \*\* P<0.0049 \*\*\* P<0.00049 | \* P<0.023 \*\* P<0.0046 \*\*\* P<0.00046 | \* P<0.025 \*\* P<0.005 \*\*\* P<0.0005 |
| Spearman's test | Correlation coefficient ρ | 0.7449 | 0.3224 | 0.4890 | 0.3692 |
| | P value | 0 (\*\*\*) | 0.1084 (ns) | 0.08991 (\*) | 0.05315 (ns) |
| | Significance after Bonferroni correction | \* P<0.019 \*\* P<0.0039 \*\*\* P<0.00039 | \* P<0.0055 \*\* P<0.0011 \*\*\* P<0.00011 | \* P<0.013 \*\* P<0.0027 \*\*\* P<0.00027 | \* P<0.0061 \*\* P<0.0012 \*\*\* P<0.00012 |
