# Supplementary material for: A microtranslatome coordinately regulates sodium and potassium currents in the human heart
Source: eLife. 2019 Oct 31;8:e52654. doi: 10.7554/eLife.52654 (PMC6867827; doi:10.7554/eLife.52654)
Supplement: Supplementary file 3. — Comparison of the average number of mRNAs particles observed to be associated and the expected number based on chance alone using centroid positions and different association criteria (from touching to 67% overlap). The significance is tested with a paired t-test Bonferroni’s correction. The number of hERG1a and SCN5A mRNAs observed to be associated is significantly above that expected by chance alone for all association criteria tested while no significant differences are observed for hERG1a/RyR2, hERG1a/GAPDH and SCN5A/GAPDH associations. [file elife-52654-supp3.pptx]

## Slide 1
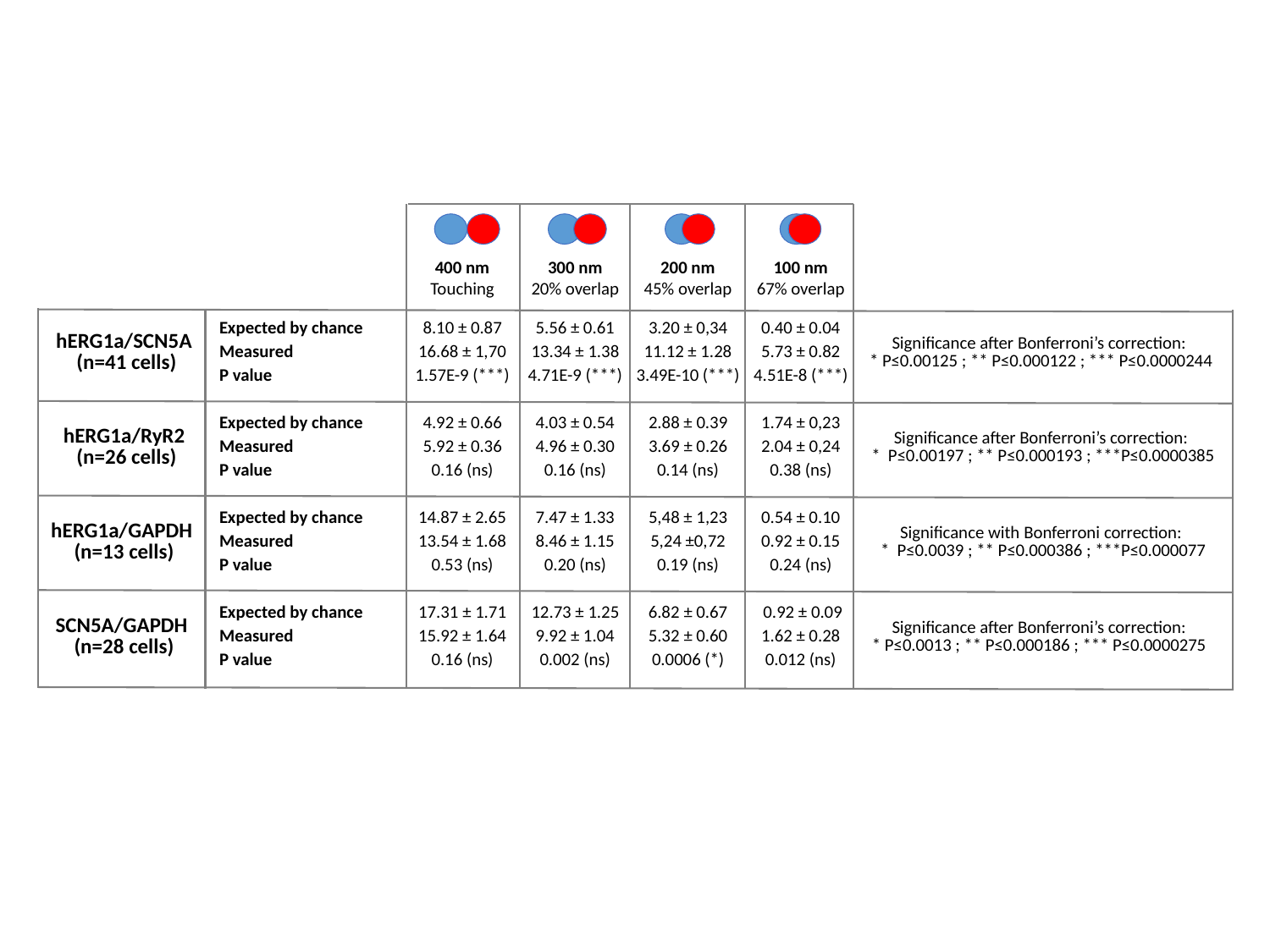

| | | 400 nm | 300 nm | 200 nm | 100 nm | |
| --- | --- | --- | --- | --- | --- | --- |
| | | Touching | 20% overlap | 45% overlap | 67% overlap | |
| hERG1a/SCN5A (n=41 cells) | Expected by chance | 8.10 ± 0.87 | 5.56 ± 0.61 | 3.20 ± 0,34 | 0.40 ± 0.04 | Significance after Bonferroni’s correction: \* P≤0.00125 ; \*\* P≤0.000122 ; \*\*\* P≤0.0000244 |
| | Measured | 16.68 ± 1,70 | 13.34 ± 1.38 | 11.12 ± 1.28 | 5.73 ± 0.82 | |
| | P value | 1.57E-9 (\*\*\*) | 4.71E-9 (\*\*\*) | 3.49E-10 (\*\*\*) | 4.51E-8 (\*\*\*) | |
| | | | | | | |
| hERG1a/RyR2 (n=26 cells) | Expected by chance | 4.92 ± 0.66 | 4.03 ± 0.54 | 2.88 ± 0.39 | 1.74 ± 0,23 | Significance after Bonferroni’s correction: \* P≤0.00197 ; \*\* P≤0.000193 ; \*\*\*P≤0.0000385 |
| | Measured | 5.92 ± 0.36 | 4.96 ± 0.30 | 3.69 ± 0.26 | 2.04 ± 0,24 | |
| | P value | 0.16 (ns) | 0.16 (ns) | 0.14 (ns) | 0.38 (ns) | |
| | | | | | | |
| hERG1a/GAPDH (n=13 cells) | Expected by chance | 14.87 ± 2.65 | 7.47 ± 1.33 | 5,48 ± 1,23 | 0.54 ± 0.10 | Significance with Bonferroni correction: \* P≤0.0039 ; \*\* P≤0.000386 ; \*\*\*P≤0.000077 |
| | Measured | 13.54 ± 1.68 | 8.46 ± 1.15 | 5,24 ±0,72 | 0.92 ± 0.15 | |
| | P value | 0.53 (ns) | 0.20 (ns) | 0.19 (ns) | 0.24 (ns) | |
| | | | | | | |
| SCN5A/GAPDH (n=28 cells) | Expected by chance | 17.31 ± 1.71 | 12.73 ± 1.25 | 6.82 ± 0.67 | 0.92 ± 0.09 | Significance after Bonferroni’s correction: \* P≤0.0013 ; \*\* P≤0.000186 ; \*\*\* P≤0.0000275 |
| | Measured | 15.92 ± 1.64 | 9.92 ± 1.04 | 5.32 ± 0.60 | 1.62 ± 0.28 | |
| | P value | 0.16 (ns) | 0.002 (ns) | 0.0006 (\*) | 0.012 (ns) | |
