# Supplementary material for: A microtranslatome coordinately regulates sodium and potassium currents in the human heart
Source: eLife. 2019 Oct 31;8:e52654. doi: 10.7554/eLife.52654 (PMC6867827; doi:10.7554/eLife.52654)
Supplement: Supplementary file 4. — Parameters were obtained after fitting to a Boltzmann equation activation and inactivation data. [file elife-52654-supp4.pptx]

## Slide 1
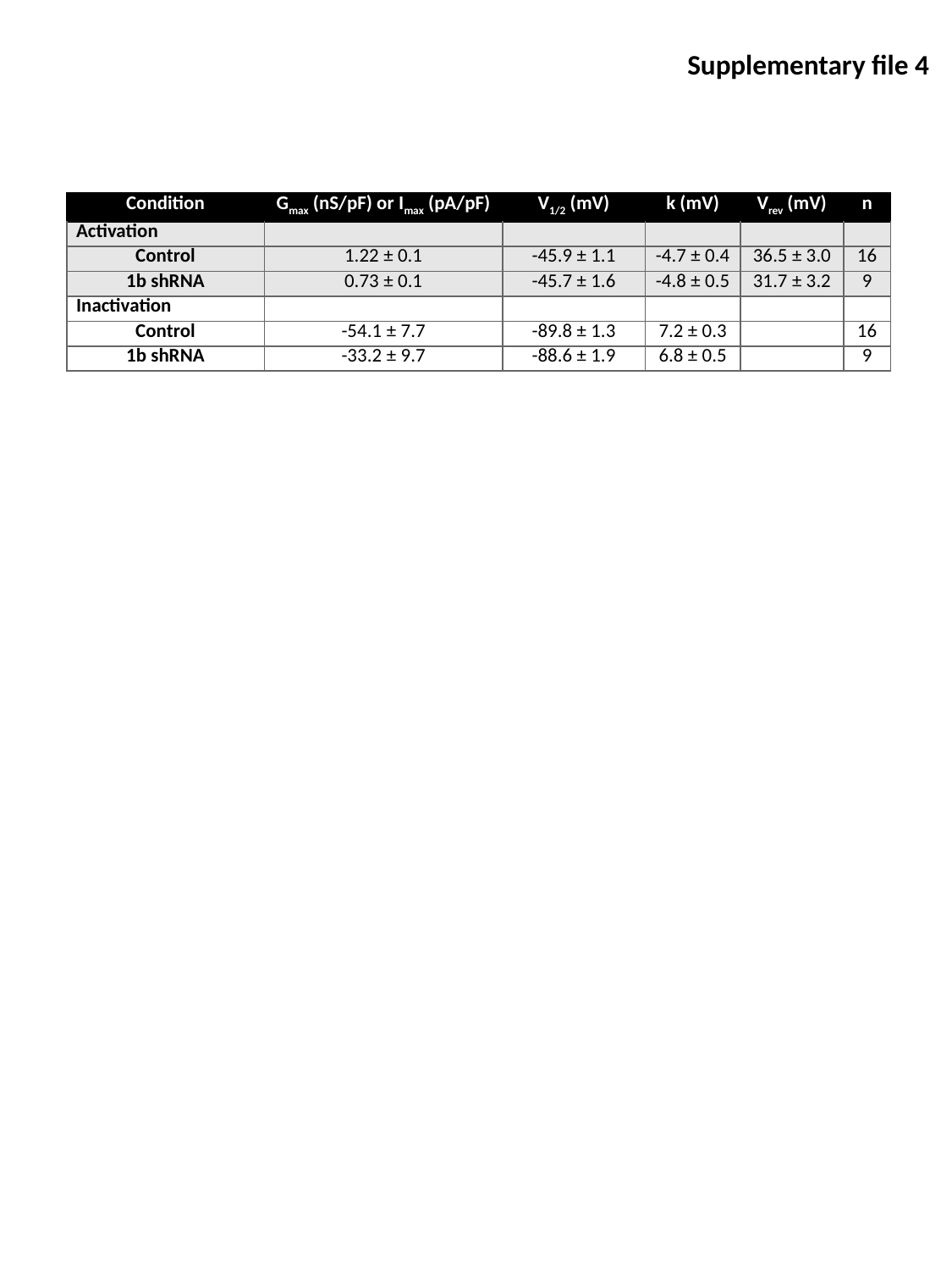

Supplementary file 4
| Condition | Gmax (nS/pF) or Imax (pA/pF) | V1/2 (mV) | k (mV) | Vrev (mV) | n |
| --- | --- | --- | --- | --- | --- |
| Activation | | | | | |
| Control | 1.22 ± 0.1 | -45.9 ± 1.1 | -4.7 ± 0.4 | 36.5 ± 3.0 | 16 |
| 1b shRNA | 0.73 ± 0.1 | -45.7 ± 1.6 | -4.8 ± 0.5 | 31.7 ± 3.2 | 9 |
| Inactivation | | | | | |
| Control | -54.1 ± 7.7 | -89.8 ± 1.3 | 7.2 ± 0.3 | | 16 |
| 1b shRNA | -33.2 ± 9.7 | -88.6 ± 1.9 | 6.8 ± 0.5 | | 9 |
