# Supplementary figures and images for: A microtranslatome coordinately regulates sodium and potassium currents in the human heart
Source: eLife. 2019 Oct 31;8:e52654. doi: 10.7554/eLife.52654 (PMC6867827; doi:10.7554/eLife.52654)

## Slide 1
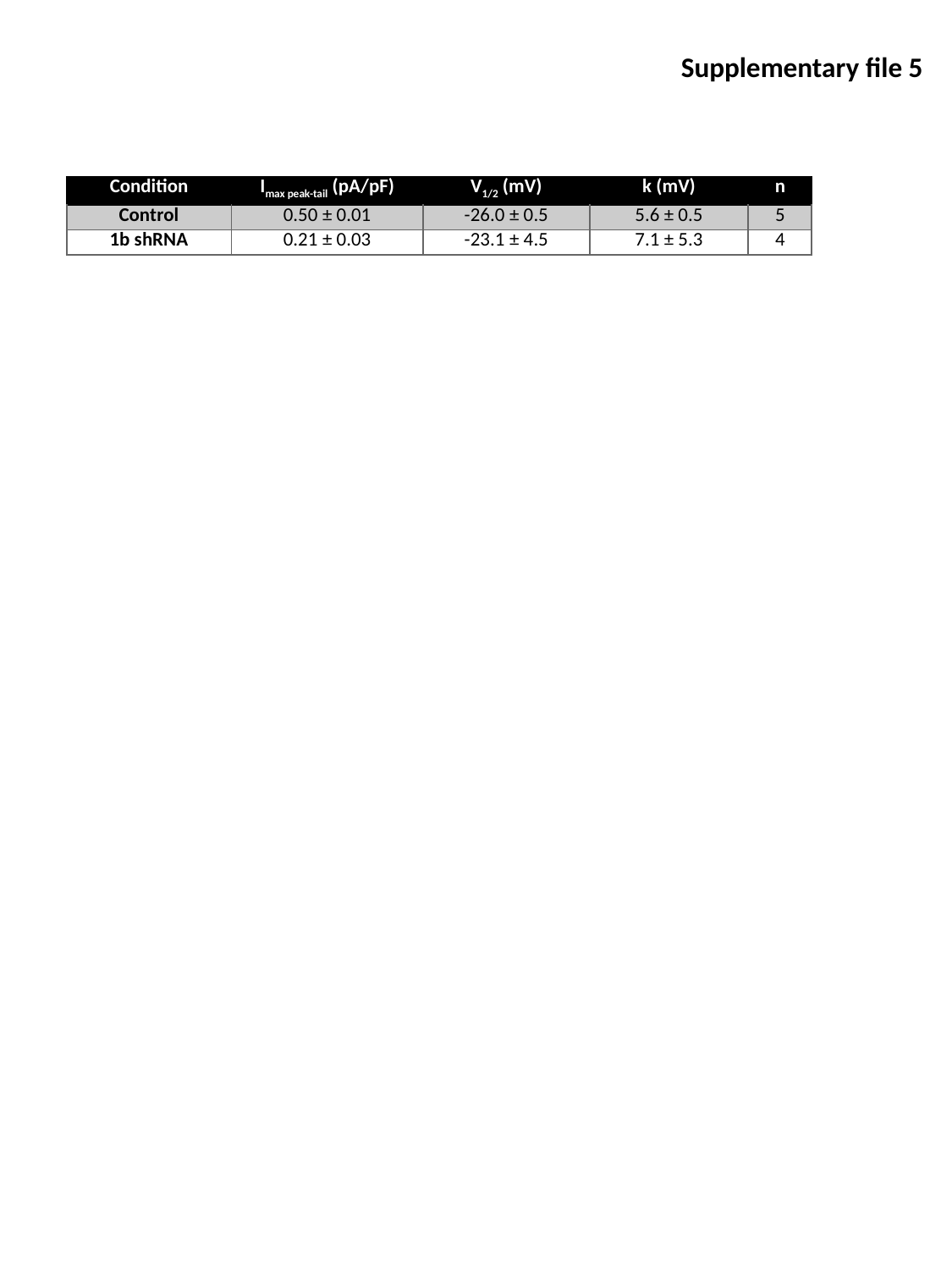

Supplementary file 5
| Condition | Imax peak-tail (pA/pF) | V1/2 (mV) | k (mV) | n |
| --- | --- | --- | --- | --- |
| Control | 0.50 ± 0.01 | -26.0 ± 0.5 | 5.6 ± 0.5 | 5 |
| 1b shRNA | 0.21 ± 0.03 | -23.1 ± 4.5 | 7.1 ± 5.3 | 4 |

Supplement: Supplementary file 5. — Parameters were obtained by fitting the experimental data of the I-V curve of the peak tail IKr to a Boltzmann equation. [file elife-52654-supp5.pptx]
